# New data on the *Paederus biacutus* species group from mainland China (Coleoptera, Staphylinidae, Paederinae)

**DOI:** 10.3897/zookeys.419.7764

**Published:** 2014-06-24

**Authors:** Zhong Peng, Li-Zhen Li, Mei-Jun Zhao

**Affiliations:** 1 Department of Biology, College of Life and Environmental Sciences, Shanghai Normal University, Shanghai, 200234, P. R. China;

**Keywords:** Coleoptera, Staphylinidae, *Paederus*, new species, new records, mainland China

## Abstract

*Paederus jianyueae* Peng & Li, **sp. n.** (Zhejiang: Qingliangfeng) is described and illustrated. Additional records of *P. biacutus* Li, Zhou & Solodovnikov, 2014 and *P. parvidenticulatus* Li, Zhou & Solodovnikov, 2014 are reported.

## Introduction

The *Paederus* fauna of China previously comprised 36 species, some of which were assigned to subgenera and some are listed as *incertae sedis* (Smetana 2004; Li et al. 2013, 2014). Five species, all of them brachypterous and more or less locally endemic, were placed in the *Paederus biacutus* species group: *Paederus biacutus* Li & al., 2014 (Fujian), *Paederus parvidenticulatus* Li & al., 2014 (Guizhou), *Paederus sinisterobliquus* Li & al., 2014 (Hubei), *Paederus symmetricus* Li & al., 2014 (Guizhou, Guangxi), and *Paederus volutobliquus* Li & al., 2014 (Guangdong). This group can be easily distinguished from other groups or subgenera of *Paederus* Fabricius, 1775 by the special color pattern (black head, elytra and abdomen; brownish red pronotum; elytra with weakly or distinctly metallic hue), four regularly arranged protrusions on the anterior margin of the labrum, the trapeziform elytra with weakly pronounced humeral angles, the notched posterior margin of the male sternite IX, the strongly sclerotized and more or less symmetrical aedeagus with a hooked or straight apex of the dorsal plate, and a pair of distinct round or triangular posterior excisions of the female sternite VIII.

A study of *Paederus* material from mainland China yielded some new records and a new species of the *Paederus biacutus* group.

## Material and methods

The morphological studies were conducted using an Olympus CX31 microscope. The images were prepared using a Canon EOS 70D (with an MP-E 65 macrolens) and Canon G12 camera. The line drawings were created using Adobe Illustrator CS3 software.

The following abbreviations are used in the text, with all measurements in millimeters:

Body length (BL): length of body from the anterior margin of the labrum to the apex of the abdomen.

Forebody length (FL): length of forebody from the anterior margin of the labrum to the posterior margin of elytra at suture.

Head length (HL): length of head from the anterior clypeal margin to the occipital constriction.

Head width (HW): maximum width of head (including eyes).

Antenna length (AnL): length of antennae from base of antennomere I to apex of antennomere XI.

Pronotum length (PL): length of pronotum along midline.

Pronotum width (PW): maximum width of pronotum.

Elytral length (EL): at the suture from the apex of the scutellum to the posterior margin of the elytra (at the sutural angle).

Elytral width (EW): maximum width of elytra.

Abdominal width (AW): maximum width of abdomen.

Aedeagus length (AL): length of the aedeagus from the apices of the parameres to the base of the aedeagal capsule.

The labels are cited in the original spelling. The type material is deposited in the Insect Collection of Shanghai Normal University, Shanghai, China (**SNUC**).

## Taxonomy

### Paederus biacutus

Taxon classificationAnimaliaColeopteraStaphylinidae

Li, Zhou & Solodovnikov, 2013

Figs 1 –2

Paederus biacutus Li, Zhou & Solodovnikov, 2013: 565.

#### Material studied

(8 ♂♂, 2 ♀♀). 1 ♂, 1 ♀, “China: Fujian Prov., Wuyishan, Guadun, 27°44'N, 117°38'E, 02.vi.2012 1,300 m, Peng & Dai leg.” (SNUC); 1 ♂, “China: Fujian Prov., Wuyishan, Guadun, Xianfengling, 27°42'N, 117°39'E, 08.iv.2013 1,200 m, Wen-Xuan Bi leg.” (SNUC); 1 ♂, “China: Fujian Prov., Wuyishan, Sangang, 27°44'59"N, 117°40'47"E, 02.vii.? 750 m, Da-Kang Zhou leg.” (SNUC); 2 ♂♂, “China: Fujian Prov., Wuyishan, Guadun, 27°44'02"N, 117°38'26"E, 30.viii.2009 1,200 m, Hao Huang leg.” (SNUC); 3 ♂♂, 1 ♀, “China: Jiangxi Prov., Yanshan County, Wuyi Shan, 27°55'45"N, 117°40'43"E, 10.v.2005 alt. 950 m, Hu & Tang leg.” (SNUC).

#### Comment.

A comparison of the original description of *Paederus biacutus* and the additional material from the type locality and its vicinity revealed some differences in the sexual characters. According to the original description and illustration, the female sternite VIII is transverse (oblong in the additional material, Fig. 2F) and the internal sac of the aedeagus has two sclerotized spines (three spines in the additional material, Fig. 2K). The previously known distribution of *Paederus biacutus* included the Chinese province of Fujian (Li et al. 2013). The above record from Jiangxi represents a new province records.

**Figure 1.**
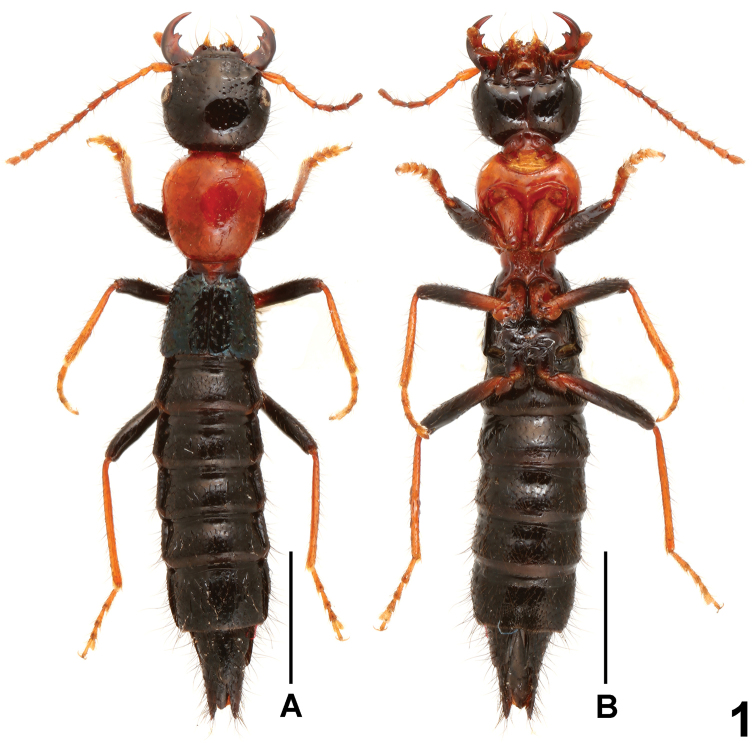
Habitus of *Paederus biacutus*. **A** lateral view **B** ventral view. Scales: 2.0 mm.

**Figure 2.**
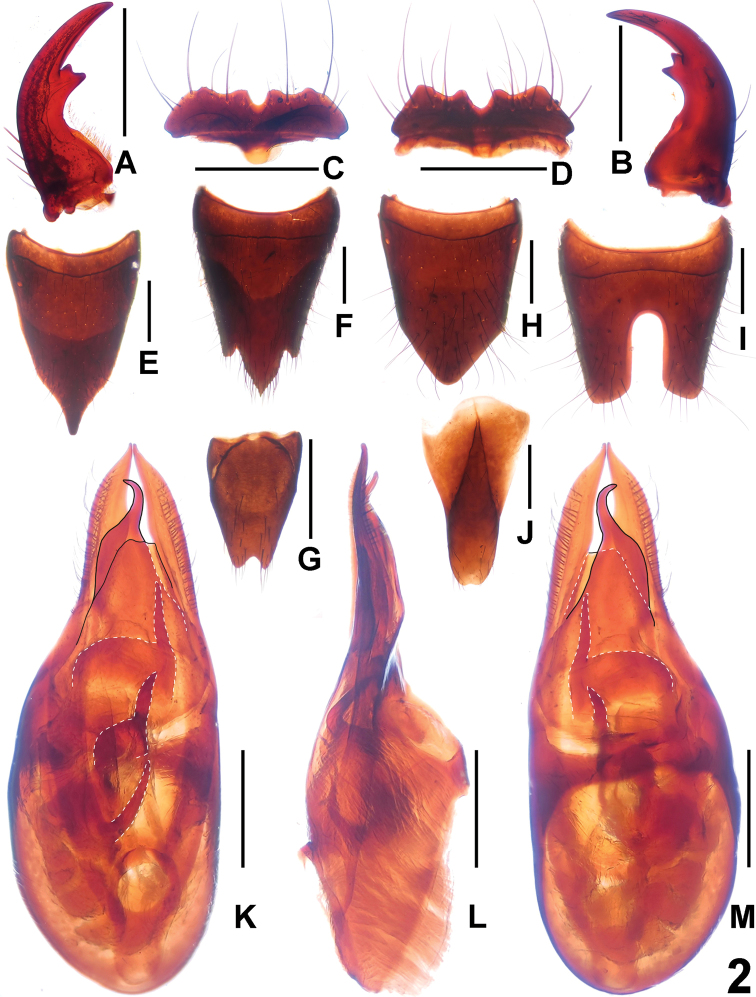
*Paederus biacutus*. **A** male left mandible **B** male right mandible **C** male labrum **D** female labrum **E** female tergite VIII **F** female sternite VIII **G** female sternite IX **H** male tergite VIII **I** male sternite VIII **J** male tergite IX **K** aedeagus in ventral view **L** aedeagus in lateral view **M** aedeagus in dorsal view. Scales: 0.5 mm.

### Paederus parvidenticulatus

Taxon classificationAnimaliaColeopteraStaphylinidae

Li, Zhou & Solodovnikov, 2013

**Figure 3.**
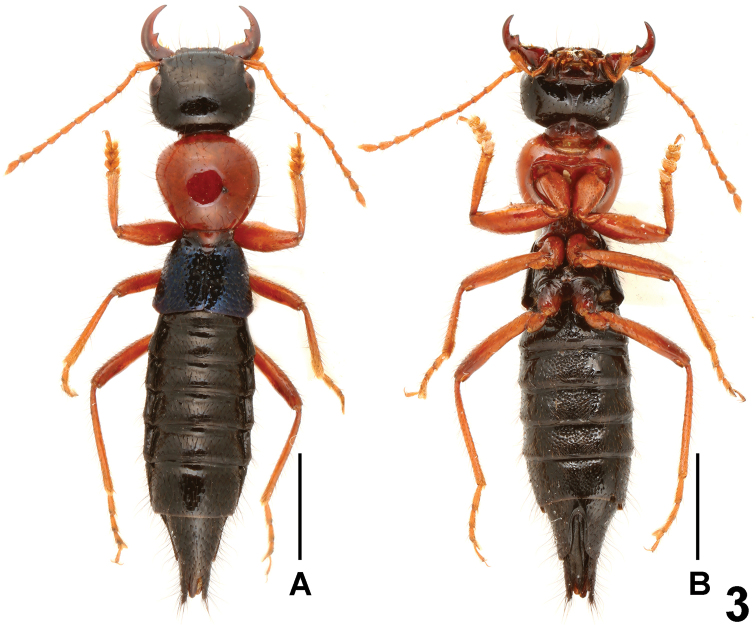
Habitus of *Paederus parvidenticulatus* (Leigong Shan). **A** lateral view **B** ventral view. Scales: 2.0 mm.

Figs 3 [Fig F4] [Fig F5] –6

Paederus parvidenticulatus Li, Zhou & Solodovnikov, 2013: 567.

#### Material studied

(16 ♂♂, 21 ♀♀). 1 ♂, “China: Guangxi Prov., Xing’an, Maoer Shan, 25°52'23"N, 110°25'06"E, 23.vii.2012 1950–2000 m, Hu & Song leg.” (SNUC); 1 ♂, 4 ♀♀, “China: Guangxi Prov., Xing’an, Maoer Shan, 25°54'23"N, 110°27'41"E, 24.vii.2012 1550–1750 m, Hu & Song leg.” (SNUC); 4 ♂♂, 14 ♀♀, “China: Guangxi Prov., Xing’an, Maoer Shan, 25°52'18"N, 110°25'01"E, 10.vii.2011 1900–2100 m, He, Tang & Peng leg.” (SNUC); 7 ♂♂, 1 ♀, “China: Guangxi Prov., Xing’an, Maoer Shan, 25°54'17"N, 110°28'04"E, 02.vi.2012 1100–1700 m, Liu & Living leg.” (SNUC); 1 ♂, 1 ♀, “China: Guizhou Prov., Leishan County, Leigong Shan, 26°22'38"N, 108°11'47"E, 06.vi.2012 1500–1600 m, Liu & Living.” (SNUC); 2 ♂♂, 1 ♀, “China: Guizhou Prov., Leigong Shan, Lianhuaping, 15.ix.2005 1450–1500 m, Li-Long Zhu leg.” (SNUC).

#### Comment.

An examination of the above material from the type localities of *Paederus symmetricus* and *Paederus parvidenticulatus* revealed that they are conspecific. A paper formally proposing the respective synonymy is being prepared by Xiao-Yan Li (pers. comm.).

**Figure 4. F4:**
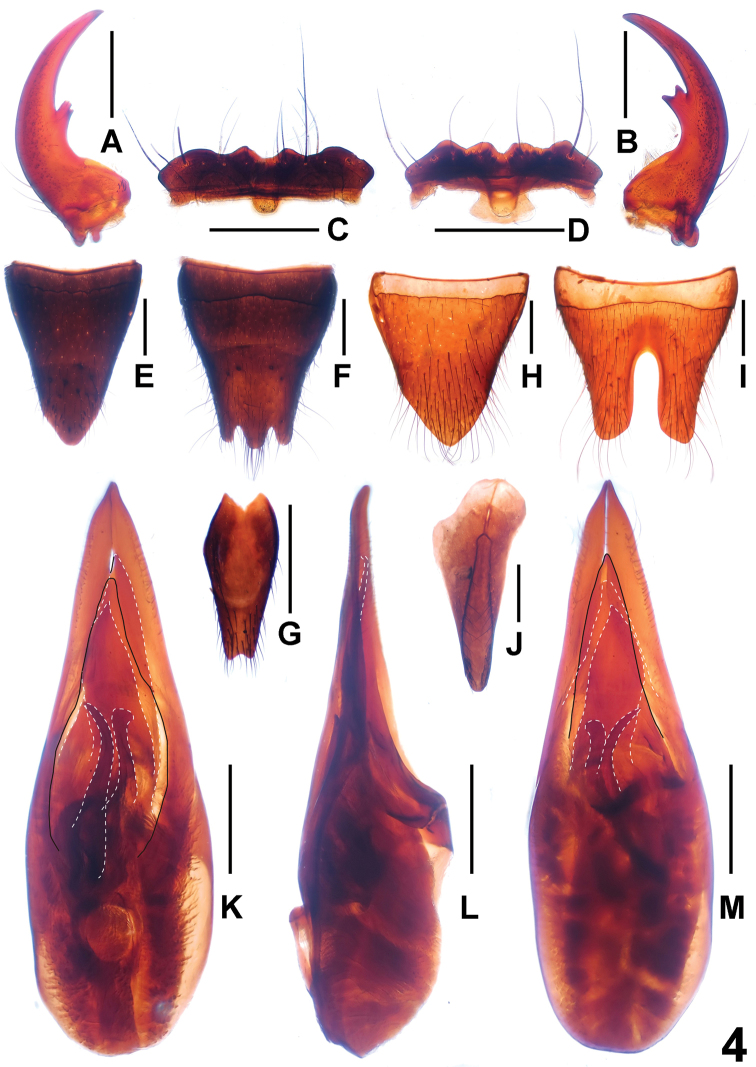
*Paederus parvidenticulatus* (Leigong Shan). **A** male left mandible **B** male right mandible **C** male labrum **D** female labrum **E** female tergite VIII **F** female sternite VIII **G** female sternite IX **H** male tergite VIII **I** male sternite VIII **J** male tergite IX **K** aedeagus in ventral view **L** aedeagus in lateral view **M** aedeagus in dorsal view. Scales: 0.5 mm.

**Figure 5. F5:**
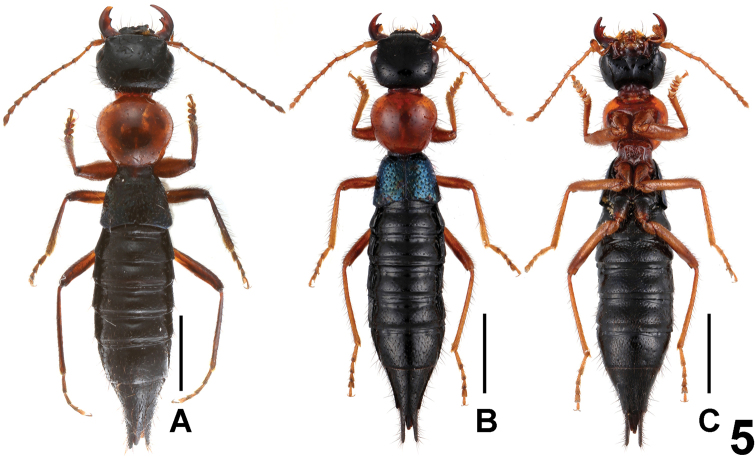
Habitus of *Paederus parvidenticulatus* (Maoer Shan). **A–B** lateral view **C** ventral view. Scales: 2.0 mm.

### Paederus jianyueae

Taxon classificationAnimaliaColeopteraStaphylinidae

Peng & Li sp. n.

http://zoobank.org/49639086-9045-438B-8153-21D951D84CAC

Figs 7 –8

#### Type material.

(14 ♂♂, 28 ♀♀). Holotype: ♂, labelled ‘China: Zhejiang Prov., Lin’an, Qingliangfeng, 30°05'48"N, 118°51'36"E, alt. 1500–1700 m 22.v.2012, Yi & Zhang leg.’ (SNUC). Paratypes: 12 ♂♂, 28 ♀♀, same label data as holotype (SNUC); 1 ♂, same data, but ‘Anhui Prov. Huang Shan, 30°07'51"N, 118°09'51"E, 18.v.2005 alt. 1700 m, Wen-Xuan Bi leg.’ (SNUC).

#### Description.

Measurements (in mm) and ratios: BL 9.23–10.34, FL 4.56–4.78, HL 1.30–1.41, HW 1.52–1.61, AnL 2.78–2.95, PL 1.59–1.67, PW 1.54–1.66, EL 1.02–1.13, EW 1.52–1.61, AW 1.70–1.85, AL 1.04–1.09, HL/HW 0.85–0.89, HW/PW 0.95–0.98, HL/PL 0.81–0.85, PL/PW 0.98–1.03, EL/PL 0.64–0.68, diameter of eye: 0.37–0.44.

Habitus as in Fig. 7. Coloration: head, pronotum and abdomen black; elytra black with faint blueish hue; legs and antennae dark-yellowish, apices of femora and tibiae not infuscate.

**Figure 6.**
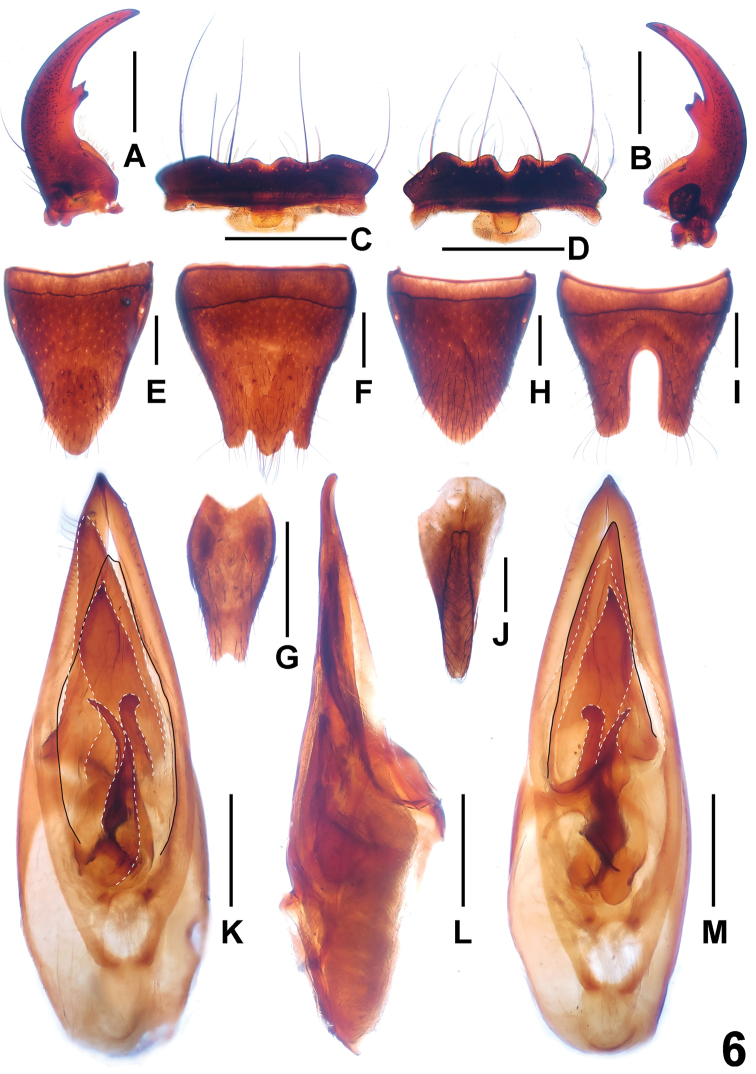
*Paederus parvidenticulatus* (Maoer Shan). **A** male left mandible **B** male right mandible **C** male labrum **D** female labrum **E** female tergite VIII **F** female sternite VIII **G** female sternite IX **H** male tergite VIII **I** male sternite VIII **J** male tergite IX **K** aedeagus in ventral view **L** aedeagus in lateral view **M** aedeagus in dorsal view. Scales: 0.5 mm.

Head transverse; shape without apparent sexual dimorphism; widest across eyes; punctation moderately coarse and very sparse; interstices glossy. Eyes distinctly convex, 0.6–0.8 times as long as postocular region in dorsal view. All antennomeres oblong.

Pronotum nearly globulous, strongly convex in cross-section; punctation similar to that of head, very sparse.

Elytra trapeziform; punctation coarse, moderately defined, and dense. Hind wings completely reduced. Metatarsomere I as long as combined length of metatarsomeres II and III.

Abdomen distinctly broader than elytra; punctation sparse; interstices with shallow microsculpture; posterior margin of tergite VII without palisade fringe; posterior margin of tergite VIII (Fig. 8E, H) strongly convex.

**Figure 7.**
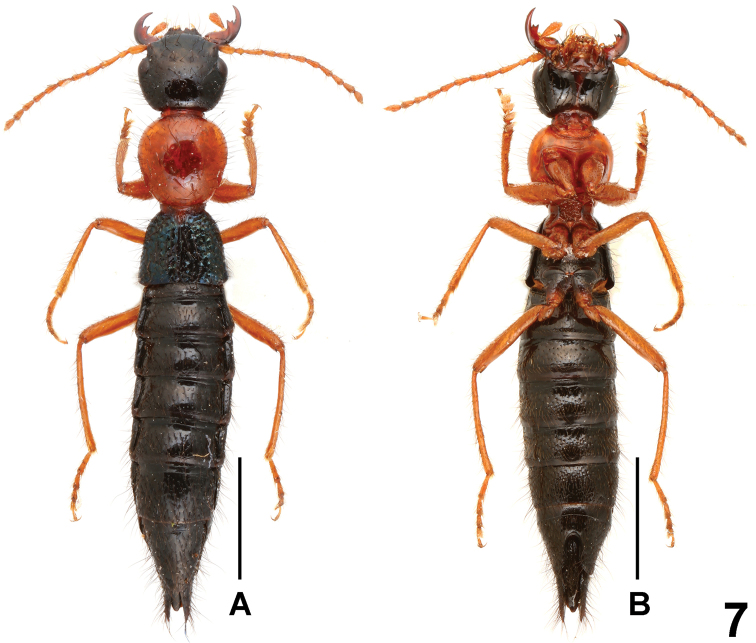
Habitus of *Paederus jianyueae*. **A** lateral view **B** ventral view. Scales: 2.0 mm.

**Figure 8.**
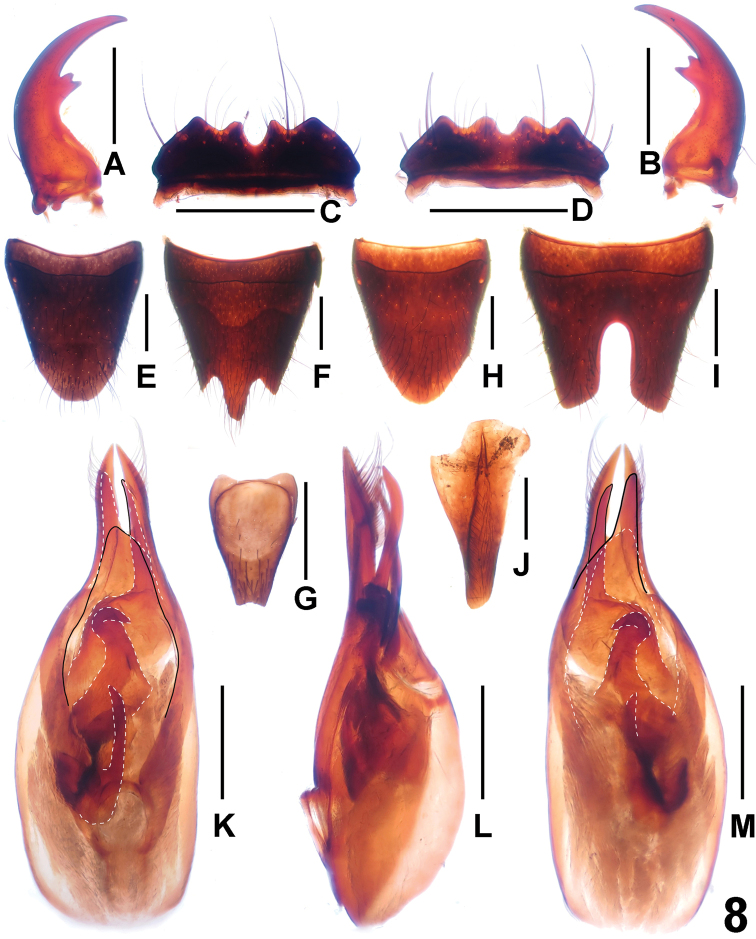
*Paederus jianyueae*. **A** male left mandible **B** male right mandible **C** male labrum **D** female labrum **E** female tergite VIII **F** female sternite VIII **G** female sternite IX **H** male tergite VIII **I** male sternite VIII **J** male tergites IX **K** aedeagus in ventral view **L** aedeagus in lateral view **M** aedeagus in dorsal view. Scales: 0.5 mm.

Male. Labrum (Fig. 8C) distinctly sinuate, anterior margin with two pairs of obtuse teeth; mandibles (Fig. 8A–B) long and robust, inner margin with one bicuspidate tooth. Sternite VII unmodified; sternite VIII (Fig. 8I) weakly transverse and with deep and narrow posterior incision, this incision approximately 0.4 times as long as sternite VIII; sternite IX (Fig. 8J) asymmetric; aedeagus as in Fig. 8K–M; dorsal plate of median lobe asymmetric, curved in lateral view and not reaching apices of parameres, its base broad and narrowed posteriad; parameres symmetric and slender; internal sac with three distinctive sclerotized spines.

Female. Labrum as in Fig. 8D. Posterior margin of sternite VIII symmetric and trifurcate as in Fig. 8F; sternite IX (Fig. 8G) symmetrical and stout.

#### Distribution and natural history.

The species was found in two geographically close localities: Qingliangfeng, western Zhejiang and the Huang Shan, southeastern Anhui. The specimens were sifted from leaf litter and moss in coniferous forests at altitudes of 1500–1700 m.

#### Etymology.

The species is named after Jian-Yue Qiu, who lent extensive support to our research.

#### Comparative notes.

*Paederus jianyueae* belongs to the *Paederus biacutus* group, as can be inferred both from the sexual characters and from the external morphology (special color pattern, four protrusions on anterior margin of labrum, morphology of the aedeagus, shape of the male sternite IX and the female sternite VIII). This new species is distinguished from other species of this group by the shape of female sternite VIII and the morphology of the aedeagus (more slender dorsal plate of the median lobe; slender parameres; three distinctive sclerotized spines in the internal sac). Based on the similar morphology of the aedeagus, *Paederus jianyueae* may be most closely related to *Paederus biacutus* Li, Zhou & Solodovnikov, 2013.

## Supplementary Material

XML Treatment for Paederus biacutus

XML Treatment for Paederus parvidenticulatus

XML Treatment for Paederus jianyueae
